# Modifying the tumour microenvironment and reverting tumour cells: New strategies for treating malignant tumours

**DOI:** 10.1111/cpr.12865

**Published:** 2020-06-26

**Authors:** Ya Qi Cheng, Shou Bi Wang, Jia Hui Liu, Lin Jin, Ying Liu, Chao Yang Li, Ya Ru Su, Yu Run Liu, Xuan Sang, Qi Wan, Chang Liu, Liu Yang, Zhi Chong Wang

**Affiliations:** ^1^ State Key Laboratory of Ophthalmology Zhongshan Ophthalmic Center Sun Yat‐sen University Guangzhou China; ^2^ Affiliated Dongguan People's Hospital Southern Medical University Dongguan China

**Keywords:** cell therapy, stem cell microenvironment, tumour microenvironment, tumour targeting

## Abstract

The tumour microenvironment (TME) plays a pivotal role in tumour fate determination. The TME acts together with the genetic material of tumour cells to determine their initiation, metastasis and drug resistance. Stromal cells in the TME promote the growth and metastasis of tumour cells by secreting soluble molecules or exosomes. The abnormal microenvironment reduces immune surveillance and tumour killing. The TME causes low anti‐tumour drug penetration and reactivity and high drug resistance. Tumour angiogenesis and microenvironmental hypoxia limit the drug concentration within the TME and enhance the stemness of tumour cells. Therefore, modifying the TME to effectively attack tumour cells could represent a comprehensive and effective anti‐tumour strategy. Normal cells, such as stem cells and immune cells, can penetrate and disrupt the abnormal TME. Reconstruction of the TME with healthy cells is an exciting new direction for tumour treatment. We will elaborate on the mechanism of the TME to support tumours and the current cell therapies for targeting tumours and the TME—such as immune cell therapies, haematopoietic stem cell (HSC) transplantation therapies, mesenchymal stem cell (MSC) transfer and embryonic stem cell‐based microenvironment therapies—to provide novel ideas for producing breakthroughs in tumour therapy strategies.

## Graphical abstract

The tumour microenvironment (TME) plays a pivotal role in tumour fate determination. TME components promote the growth, metastasis, immune escape, stemness, and drug resistance of tumour cells. Therefore, modifying the TME to effectively attack tumour cells could represent a comprehensive and effective anti‐tumour strategy. Normal cells, such as stem cells and immune cells, can penetrate and disrupt the abnormal TME. Reconstruction of the TME with healthy cells is an exciting new direction for tumour treatment.

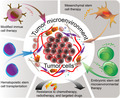

## 1 INTRODUCTION

Tumour incidence and mortality are increasing yearly, with particularly rising trends in younger populations.^1^ In 2018, 18.1 million new tumour cases were reported worldwide, and 9.6 million people died from tumours, making them one of the greatest threats to human health.^2^

The generation and development of tumours were previously believed to depend on only tumour suppressor or oncogene mutations, the basis of the "tumour‐centric" view.^3^ Therapies derived from this theory, whether drugs, surgeries or radiation therapies, are all based on killing tumour cells with inevitable secondary damage and increasing treatment resistance. Researchers have found that the tumour microenvironment (TME) plays a pivotal role in the generation, progression and metastasis of tumours. A century ago, Stephen Paget found that breast cancer metastasis displayed organ (tissue) preference, which related to the cell environment of the targeted organ (tissue). He boldly assumed that tumour progression is controlled by the interaction of tumour cells and the external environment, and first proposed the concept of the TME.^4^ Various components of the TME constitute an intricate network that precisely regulates tumour fate and the interactions of tumour cells with other components. This enables tumour cells to steadily proliferate, resist apoptosis, escape from immune elimination, maintain stemness and metastasize to distant sites. The TME theory superseded the theory that the fate of tumour cells is determined only by their genetic material and provided a new perspective for comprehensively understanding tumour metastasis and drug resistance mechanisms.

Traditional anti‐tumour chemoradiotherapy is strongly cytotoxic because it denatures nucleic acids and proteins in tumour cells; however, this also results in damage to normal cells and causes serious adverse reactions, even secondary tumour formation.^5^, ^6^, ^7^, ^8^ Tumour cells escape apoptosis by constantly generating new gene mutations that mediate tumour drug resistance. To solve the problem of the poor specificity of chemoradiotherapy, targeted therapies and immune therapies have been developed.^9^ Although immune therapies, such as anti‐programmed death 1(PD‐1)/PD‐L1 treatment, show considerable efficacy in several tumours, they still have individual specificity. Meanwhile, the high incidence of severe autoimmune adverse reactions after immune therapy poses a new threat to patients' lives.^10^, ^11^, ^12^, ^13^

With the gradual deepening of understanding of TME, targeting TME compounds to undermine protecting hotbed of tumours have become an effective means of cancer treatment. Large amount of pre‐clinic and clinic study proved the quietly success in targeting angiogenesis, extracellular matrix (ECM) and cells components within TME.^14^ In recent years, cell therapies are fast rising and have been proven to have powerful functions and ensured safety. Compared with the single role of drug, cells may act on TME from multi‐angle and through many ways at one time due to its better plasticity. It is manifested that cell therapies can inhibit or reverse tumours for which there is currently no effective therapy. We suggest that utilizing a therapeutic cell's own microenvironment to regulate and modify the TME, thereby destroying the tumour nests that tumour cells depend on for survival, constitutes a new direction for tumour treatment. We will elaborate on the current therapies, especially cell therapies, for targeting tumours and the TME—such as immune cell therapies, stem cell replacement therapies (mainly used for bone marrow‐derived tumours), mesenchymal stem cell (MSC) transfer and embryonic stem cell‐based microenvironment therapies—to provide novel ideas for the optimization of tumour therapy strategies.

## 2 COMPOSITION AND FUNCTION OF THE TME

The components surrounding the tumour cells constitute the functional TME in which tumour cells initiate and grow and from which they invade and metastasize. The TME is a sophisticated network that includes various tumour‐associated cells, such as cancer‐associated fibroblasts (CAFs), cancer stem cells (CSCs), MSCs, tumour‐associated immune and inflammatory cells, pluripotent stromal cells, cancer‐associated adipocytes, pericytes and endothelial cells (ECs). The tumour‐associated cells secrete tumour‐associated exosomes (TEXs) and soluble molecules, including cytokines, kinases, protein, transcription factors, growth factors, hormones and free radicals. They regulate and respond to each other, construct the scaffold of the tumour ECM, and are nourished by tumour angiogenesis, manifesting as a special niche for innutrition, acidity, hypoxia and ischaemia.^15^, ^16^ The TME has a degree of tumour‐derived organ and tissue specificity, reviews of Schumacher et al^17^ also summarized that human tumours vary substantially in the composition of their microenvironment, and this is likely to regulate cancer cell morphology and influence the ability of the individual T‐cell immunotherapy.

### 2.1 The ECM forms the 3‐dimensional tumour protective nest

The tumour ECM, comprising collagen, fibronectin, laminin, vitronectin and tenascin, is secreted by cells in the TME. Via autocrine and paracrine signalling, tumour cells and microenvironment cells, especially CAFs, transform the ECM to an advantageous phenotype for tumour progression.^18^ Collagen and fibronectin in the ECM provide physical support for tumour cells, and proteoglycans act as binding factors for growth factors and cytokines.^16^ Thus, the ECM not only provides a 3‐dimensional structure for tumour growth, but, more importantly, it provides precise biomechanical and biochemical regulation for tumour cells.^19^

The alteration of the orderly isotropic arrangement of the collagen, called matrix remodelling, results in tumour‐associated collagen signatures (TACS).^20^ Different TACS stages correspond to different degrees of tumour progression. Over activated and transformed CAFs induce type I collagen aberrations, transforming type I TACS to type III, which manifests as fibre thickening, cross‐linking, deposition, distribution perpendicular to the tumour boundary, developing tensile stresses and formation of a tumour invasion track.^21^ This growing fibrosis and stiffness of ECM results in tissue “desmoplasia,” which stimulate tumorigenesis, metastasis and immune surveillance.^22^, ^23^ Furthermore, the ECM stiffness is obstructive for effective permeation of drugs to the intratumoral area and promotes transformation of cancer cells to CSCs, and these two aspects can induce resistance to anti‐cancer therapies.^24^ Whatcott et al^25^ demonstrated that high desmoplasia is related to low survival rates of pancreatic ductal adenocarcinoma patient, indicating the reprogramming in the tumour stroma is most effective for targeting desmoplastic tumours, such as pancreas.^26^

### 2.2 Angiogenesis causes hypoxia and low drug penetration while promoting tumour immune escape and metastasis

The infinite proliferation and malignant metastasis of solid tumours are inseparable from angiogenesis in the TME. Tumour angiogenesis is the formation of abnormal vascular tissue with an immature structure, abnormal function and high permeability; it contributes to abnormal haemodynamics, poor nutrient, and oxygen supply, and TME hypoxia and hypoperfusion. Hypoxia is a common condition in many solid tumours. The hypoxic TME harbours cells with a burst of proliferation activity, effective for overgrowing a mass of tumour.^27^ It has been identified a bidirectional cross‐linking between hypoxia and angiogenesis so that the activity of either one potentiating another.^28^, ^29^ Tumour angiogenesis also results in low intratumoral drug concentrations and inhibits immune cell infiltration of the TME, thereby laying the foundation for drug resistance, immune escape and metastasis.^30^ Vascular endothelial growth factor (VEGF) is believed to be the most important factor for angiogenesis, a process which depends on the activation, germination and tube formation of ECs.^31^, ^32^ Most cells in the TME, including tumour cells, can activate vascular ECs by secreting VEGF. This prompts the vascular ECs to accelerate proliferation and stretch out more pseudopodia to enhance their signal capture and invasion capabilities, which initiates angiogenesis. In addition to VEGF, matrix metalloproteinase (MMP) 2/9, transforming growth factor α/β (TGFα/β), fibroblast growth factor (FGF) and platelet‐derived growth factor (PDGF) in the TME can also promote tumour angiogenesis.^33^

### 2.3 Cells in the TME are core links in the signal communication network

The cells in the TME are collectively referred to as tumour‐associated stromal cells; they regulate tumour fate through complex signal communication with tumour cells. Tumour‐associated stromal cells include CAFs, tumour‐associated ECs, pericytes, CSCs, MSCs and immune cells, and other rarer stromal cell populations, such as neurons, fibrocytes, adipocytes and follicular dendritic cells, have also been observed in some tumours.

#### 2.3.1 CAFs

Normal fibroblasts and adipose‐derived stem cells in the TME are transformed into CAFs that overexpress α‐smooth muscle actin (α‐SMA) and fibroblast activation protein.^34^ CAFs occupies approximately 80% of pancreatic and breast tumour volume, and from cellular view, CAFs take about 40%‐50% of the whole population of cells within the tumour microenvironment.^26^ The secretory capacity of CAFs is much greater than that of normal stromal cells, and CAFs are the main source of tumour cell ECM.^35^ CAFs and tumour cells reciprocally regulate each other through a feedback mechanism. CAFs represent metabolic symbiosis with cancer cells, which is important therapeutically.^36^, ^37^ TGFβ1, PDGF, FGFβ and connective tissue growth factor secreted by tumour cells promote the transformation of normal fibroblasts into CAFs. TGFβ, FGF and hepatocyte growth factor (HGF) secreted by CAFs act on tumour cells, inducing epithelial‐mesenchymal transition (EMT) and a pre‐metastatic state.^38^, ^39^, ^40^ The matrix proteins secreted by CAFs differ in composition and content from those produced by normal tissues, forming the TME‐specific ECM stiffness. However, CAFs also play an important role in matrix degradation by secreting matrix enzymes such as MMPs, which engages cancer cells to reshape their morphology and breakthrough basement membrane. Both deposition and degradation of ECM induced by CAFs are cancer promoting and CAFs could be essential target for cancer therapies.^41^

In addition, CAFs interplay with other stromal cells to regulate TME ecology. CAFs' secretomes reprogramme cancer cells, immune cells and ECs to facilitate cancer cell invasion and metastasis. Increased glycolysis of CAFs creates acidity in TME to increase generation of myeloid‐derived suppressor cells (MDSCs), inhibit maturation of tumour‐associated dendritic cells (TADCs) and impair the activity of NK and effector T cells.^15^ As the most population of stomal cells, CAFs accumulation in the TME is often correlated with poor prognosis in many tumours.^42^

#### 2.3.2 Immune cells

**FIGURE 1:**
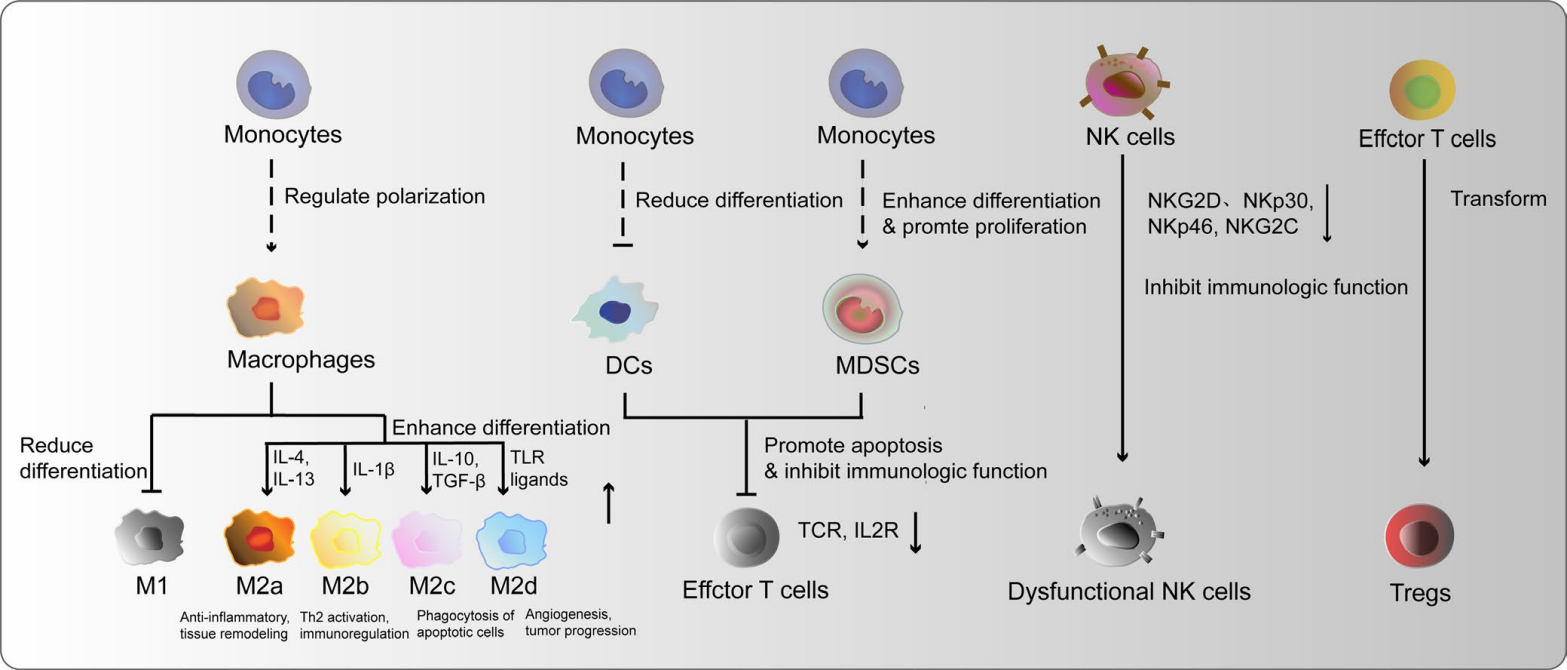
Abnormal and inactivated immune cell populations in the TME. Monocytes in the TME are prompted to differentiate into tumour‐supporting M2 macrophages and MDSCs, whereas their differentiation into M1 macrophages and DCs is impaired. The presence of fewer DCs and more MDSCs results in the inhibition of effector T‐cell responses through the down‐regulation of the TCR and IL‐2R. NK cells in the TME are inhibited through the down‐regulation of their expression of NK activating receptors, such as NKG2D, NKp30, NKp46 and NKG2C. Traditional effector T cells transform into Tregs that have tumour protective effects. DC, dendritic cell; IL‐2R, interleukin‐2 receptor; MDSC, myeloid‐derived suppressor cell; NFκB, nuclear factor kappa‐B; NK, natural killer; TCR, T‐cell receptor; TME, tumour microenvironment; Tregs, regulatory T cells

Both innate and adaptive immune cells in the TME show low tumour‐recognition and killing ability, which is a key factor contributing to tumorigenesis and progression (Figure 1).

The TME affects innate immune cells in various ways. (a) Tumour‐associated macrophages (TAMs) are mainly generated from bone marrow‐derived monocytes or tissue‐resident macrophages and are tumour‐promoting by participating in tumour angiogenesis and metastasis. TAMs are highly heterogeneity and recently identified as a spectrum concept for macrophages activation based on the stimuli and secreted cytokines and chemokines. M1 macrophages, stimulated by LPS and interferon gamma, are identified by their production of high levels of pro‐inflammatory cytokines and promotion of the Th1 response in the adaptive immune response. M2 macrophages are activated by IL‐4 or IL‐13 ligands and notably polarized into M2a (anti‐inflammatory, tissue remodelling), M2b (Th2 activation, immunoregulation), M2c (phagocytosis of apoptotic cells), M2d (angiogenesis, tumour progression) subtypes, which are response for tumour progression and the main population of macrophages in TME.^43^ IL‐10, IL‐4, IL‐13, CCL2, CCL3, CCL4, CCL5 and MFG‐E8 in TME promote M2 macrophages generation. TAMs participate in the tumour angiogenesis by secreting pro‐angiogenic factors, including VEGF‐A, EGF, PlGF, TGF‐β, TNF‐α, IL‐1β, IL‐8, CCL2, CXCL8 and CXCL12, and enable tumour metastasis by up‐regulated N‐cadherin and Snail, whereas down‐regulated E‐cadherin.^44^ Furthermore, M2 subtype TAMs regulate the emergence of regulatory immune‐inhibiting cells by secreting abundant anti‐inflammatory cytokines.^45^ (b) Signalling molecules in the TME, such as MHC class I chain‐related protein A and B, drive low expression of activating receptors like NKG2D, NKp30, NKp46 and NKG2C in natural killer (NK) cells, thereby inhibiting their anti‐tumour effects.^46^, ^47^ (c) Dendritic cells (DCs) present antigen to T cells through MHC molecules and recruit other immune cells by secreting pro‐inflammatory cytokines and chemokines. Anti‐inflammatory factors in the TME disrupt the normal differentiation of monocytes into DCs and enhance transformation of immature regulatory DCs (RegDCs). RegDCs can inhibit T‐cell function by secreting TGFβ and prostaglandin E_2_.^48^ (d) MDSCs generated by abnormal differentiation of monocytes. It secretes S100A8/A9, which enhance tumour cell survival, and can inhibit T‐cell responses by secreting TGFβ, raising intracellular reactive oxygen species (ROS) and l‐arginine level, and activating the IL‐10 pathway.^49^ MDCSs act together with TAMs to remodelling ECM, promoting angiogenesis and metastasis.^50^

Adaptive immune cells in the TME are altered to promote tumour immune escape. The expression of the T‐cell receptor (TCR) and interleukin‐2 receptor (IL‐2R) are down‐regulated in otherwise immunocompetent CD8+ T cells, resulting in immune suppression. Stomal cells, such as BECs, pericytes, CAFs and MSCs, express PD‐L1, T‐cell immunoglobulin and mucin‐containing domain molecule 3 (TIM3) and CD95L (also known as FASL) on their surface, and they secrete indoleamine 2,3‐dioxygenase (IDO), interleukin‐6 (IL‐6), prostaglandin E2 (PGE2) and transforming growth factor‐β (TGFβ) to induce exhausting and anergy of effector T cells.^16^ Meanwhile, expression of members of the FAS/FASL and PD‐1/PDL‐1 pathways is up‐regulated in the anomalous T cells, whereas the PI3K/AKT pathway is down‐regulated, which inhibits T‐cell proliferation and induces apoptosis.^51^, ^52^ In addition, regulatory T cells (Tregs), which are CD4^+^CD25^high^FOXP3^+^, are induced from traditional T cells trough TGFβ1 and IL‐10 signalling by TAMs, RegDCs and other non‐immune cells. NK cells and cytotoxic T Lymphocytes (CTLs) play a key role for elimination of malignant tumour. The two cell types show metabolic interdependencies with tumour cells, which causing metabolic competition, nutrient restriction and immunosuppression.^53^ M2 macrophages, CAFs and Tregs make immunologic restrains against CD8+ T cells, thus causing CTLs dysfunction and exhaustion. Therefore, the T cells lose their normal functions and even mediate some of the major tumour protective effects in the TME.^54^ Recalling immunity cycle in tumour through priming and activation of effector T cells is the key to exert durable and fruitful effects against tumour.^55^

#### 2.3.3 Tumour‐associated ECs

Tumour‐associated ECs include blood endothelial cells (BECs) and lymphatic endothelial cells (LECs). VEGFA in the microenvironment promotes the formation of new blood vessels from BECs, and VEGFC and VEGFD activate LECs to form new lymphatic vessels in the TME.^56^ Low expression of E‐selectin, intercellular adhesion molecule (ICAM) 1/2 and vascular adhesion molecule 1 (VCAM1) in tumour‐associated ECs results in the loss of tight connections in the tumour vascular endothelium. This leads to vascular structural abnormalities and hemodynamic disorders, inhibits the recruitment of immune cells and induces CD8^+^ T‐cell immune tolerance by up‐regulating the expression of programmed death‐ligand 1/2 and the CD28‐CTLA4 family receptor ligands B7‐H3 and B7‐H4.^57^ The leaky vasculature also causes precipitation and accumulation of waste products, and promotes TME acidity that influences tumour metabolism.^58^ The acidified TME drives tumour local invasion.^59^

#### 2.3.4 Pericytes

Pericytes differentiate from mesenchymal precursors and are recruited to tumours by platelet‐derived growth factor‐β (PDGFβ) gradients.^60^ Pericytes are heterogenous in their function and are coming into focus these years. Nestin+/NG2+ “Type‐2” pericytes contribute to tumour angiogenesis by promoting ECs survival through their secretomes including VEGFA, ANGPT1 and ECM components. They also express neural cell adhesion molecule 1 (NCAM1) and the NG2 proteoglycan, which contribute to vascular maturation by increasing pericyte recruitment.^61^ Furthermore, pericytes are involved in CSCs maintenance, tumour metastasis and immune microenvironment. Upregulation of PD‐L1, CD90, PDGFRβ, CD248 and Rgs5 which inhibit CD4+ and CD8+ cytotoxic T‐cell activity was reported in pericytes derived from within tumour microenvironments, which facilitates immunosuppression and eventual immune evasion of tumour cells.^62^

#### 2.3.5 MSCs

During the progression of carcinogenesis, MSCs are recruited to the TME by growth factors, cytokines and chemokines, such as IL‐6, IL‐1β, TGF‐β1, EGF, PDGF, TNF‐α and SDF‐1α, secreted by cancer cells and stomal cells.^63^ Upon entry into the tumour microenvironment niche, MSCs promote or inhibit tumour progression by various mechanisms. The anti‐tumour effect of MSCs is largely dependent on their immune‐activating function: MSCs could enhance the phagocytic ability of co‐cultured macrophages, and TLR‐3 activated MSCs preserves viable and functional neutrophils by amplifying the antiapoptotic effects. MSCs have the ability to stimulate resting T cells to become activated and to proliferate, and can behave as conditional DCs in syngeneic immune responses by secreting pro‐inflammatory cytokines. Many researches also state the pro‐tumour effect of MSCs: once homing to TME, MSCs could differentiate into several stomal cells, such as CAFs, pericytes, adipocytes and endothelial‐like cells to participate in TME construction and evolution.^64^, ^65^ MSCs promotes tumour growth by increasing the number of cancer stem cells through bone morphogenetic protein signalling and WNT, TGF‐β, IL‐6/JAK2/STAT3 signalling pathways.^66^

### 2.4 TEXs are TME messengers

Exosomes are secreted vesicle‐like membrane structures with a diameter between 30 and 100 nm. They carry a variety of proteins, lipids and nucleic acids, and contribute to intercellular communication.^67^ TEXs, as the microcosms of tumour cells, carry a large amount of tumour‐derived materials with source‐cell specificity; TEXs are one of the main methods of TME signal interaction. TEXs participate in almost all tumour processes, including angiogenesis, matrix remodelling, tumour metastasis and immune evasion.

TEXs regulate tumour angiogenesis by activating ECs.^68^ Pancreatic cancer‐derived CD106^+^CD49d^+^ TEXs are recruited, recognized and internalized by tumour‐associated ECs. They induce the expression of VEGF and other angiogenic proteins, such as CXCL5, MIF, and CCR1, by ECs and promote angiogenesis in the TME.^69^ In addition, many non‐coding RNAs in TEXs have been shown to play a key role in angiogenesis. Non‐coding RNAs (including miR‐9, miR‐21 and miR‐210) in TEXs can promote angiogenesis in lung cancer by activating STAT3, ephrin A3 and MMP2/9.^70^, ^71^, ^72^, ^73^ Conigliaro et al found that exosomes secreted by CD90^+^ liver cancer cells are rich in long non‐coding RNA (lncRNA) H19, which can up‐regulate the expression of VEGF, VEGF receptor and ICAM in vascular ECs, thereby promoting tumour angiogenesis and tumour cell adhesion and migration to the site of neovascularization. This finding suggests that lncRNA H19 may also be related to haematological metastasis of tumours.^74^ Lang et al^75^, ^76^ confirmed that lncRNA CCAT2 and lncRNA POU3F3 in glioma‐derived exosomes are related to angiogenesis in vivo. Meanwhile, TEXs derived from various tumours—such as melanoma,^77^ chronic myeloid leukaemia,^78^ glioma,^79^ and breast,^80^, ^81^ colon,^82^ and ovarian cancer^83^ have been shown to promote angiogenesis in the TME.

Matrix enzymes in TEXs degrade normal ECM, promote matrix remodelling and create a pre‐metastasis microenvironment.^84^, ^85^ For example, melanoma‐derived exosomes can promote glycolysis in skin dermal cells, inhibit oxidative phosphorylation and acidify the ECM through their delivery of miR‐155 and miR‐210 to prepare for tumour metastasis.^86^

TEXs disrupt the immune function of tumour patients and promote immune escape of tumour cells. They inhibit the anti‐tumour activities of monocytes, DCs, T cells, macrophages, and NK cells, and drive the development of MDSCs and Tregs to promote tumour immune tolerance.^52^, ^87^, ^88^

Manipulation of extracellular vesicles (EVs) to carry a desired cargo is a novel strategy for tumour therapy. EVs can be modified with specific receptors so as to target the cell/s of interest; this will pursue a long‐term content storage, virtue by no phenotypical alteration inside the TME. This approach is impressive and covers the current limitation for application of stem cells for cancer therapy due to encountering phenotypical alterations in the TME. Another impressive feature with EVs therapy is their stability and their capacity to cross biological barriers efficiently.^89^

## 3 TME‐MEDIATED TUMOUR PROTECTION AND PROMOTION MECHANISMS

**FIGURE 2:**
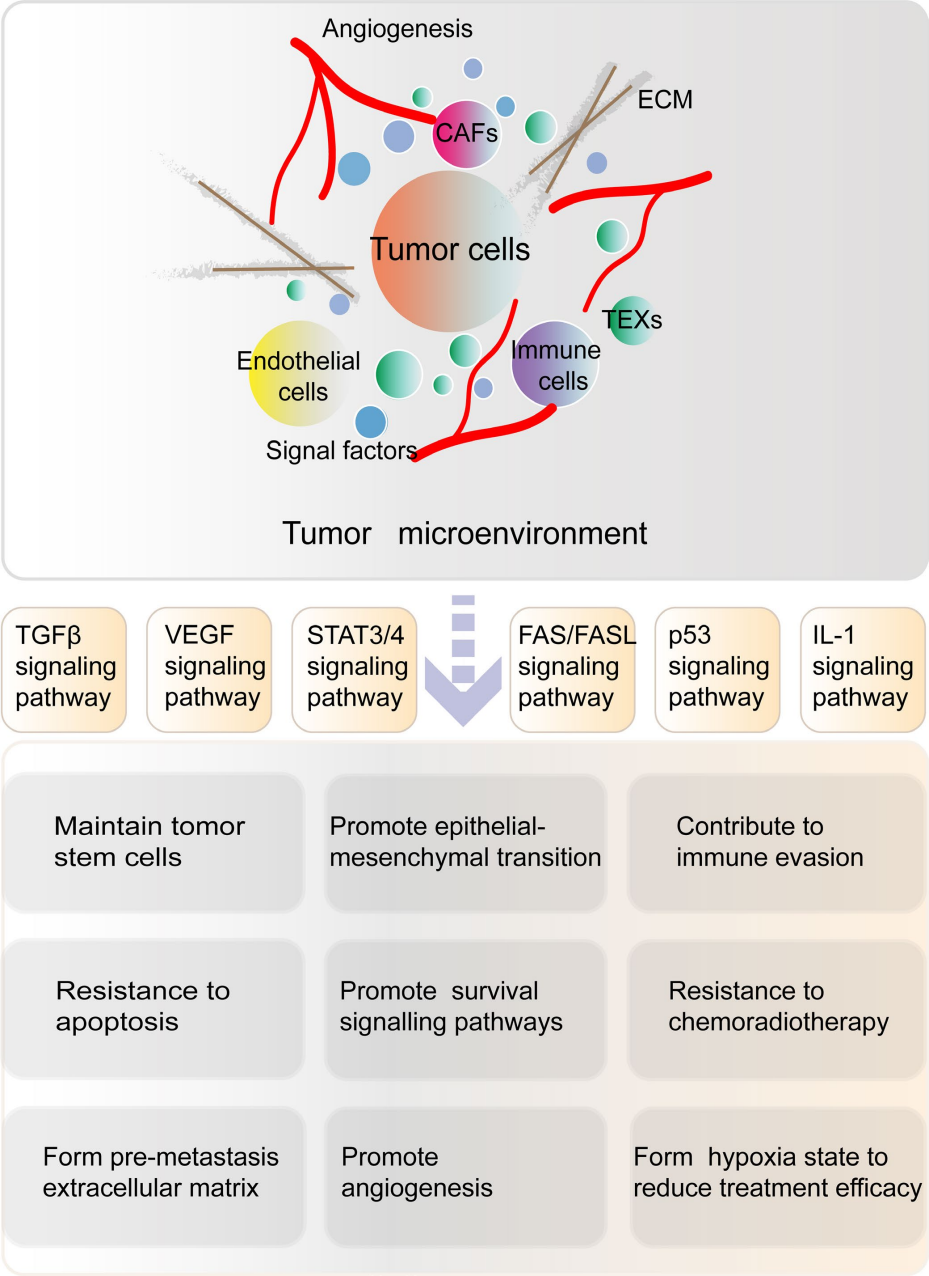
Composition of the TME and its effects on tumour development. The TME is a complex network of tumour‐associated cells (such as CAFs, tumour‐associated immune cells, and ECs), TEXs, soluble molecules (such as cytokines, growth factors and hormones) and tumour‐specific ECM, which is nourished by tumour angiogenesis. The components act as a tumour nest that maintains CSCs, promotes EMT, enables escape from immune surveillance and provides resistance to therapies through specific signal pathways. CAF, cancer‐associated fibroblast; CSCs, cancer stem cells; ECM, extracellular matrix; ECs, endothelial cells; EMT, epithelial‐mesenchymal transition; TEX, tumour‐associated exosome; TME, tumour microenvironment

Various parts of the TME act as tumour hotbeds to promote their malignancy (Figure 2). To aid the search for effective anti‐tumour targets, we will consider the role of the TME in maintaining the tumour reserve, promoting tumour metastasis and resisting killing from 2 angles: (a) promotion of CSCs generation and (b) initiation of EMT.

### 3.1 The TME protects CSCs

CSCs are a relatively quiescent tumour cell subpopulation that undergoes active DNA repair, similar to normal adult stem cells. As the "foundation" of tumours, CSCs mediate therapy resistance and metastasis of tumours; they make a tumour "the endless weed under wildfire."^90^

Oxygen depletion by highly proliferative cancer cells and dysfunctional tumour angiogenesis leads to microenvironmental hypoxia. CSCs are concentrated in the hypoxic niche in which hypoxia and hypoxia‐inducible factors (HIFs), mediators of hypoxia, induce their resistance by promoting metabolic reprogramming, stem cell maintenance and tumorigenesis. Hypoxia and HIFs could maintain stemness of CSCs by induction of EMT, activation of stemness‐related signalling pathways, activation of stemness‐related genes (Sox2, Oct4, Nanog, BMI1, MYC and KLF4), suppression of differentiation‐related genes and so on. CSCs in hypoxic TME uptake more glucose and reprogramme their metabolic mechanism from oxidative phosphorylation to glycolysis, which is responsible for resistance to radiation and chemotherapy and promoting DNA repair.^91^ 31734836 One of the key features of CSCs is the evolving of drug efflux pumps which are ATP‐binding cassette (ABC) transporters. These are unidirectional cellular pumps that cause their resistance. The efflux pumps reduce accumulation of drugs within the stem cells and disable the activity of the chemotherapeutic drugs. The activity of efflux system is potentiated by hypoxia.^28^, ^92^, ^93^ Furthermore, hypoxia and HIFs induce dedifferentiation and enhances telomerase activity of CSCs to endow them resistance.^93^ Hypoxia‐induced stem phenotypes have been confirmed in many types of tumours. Hypoxia can increase the expression of the stem cell markers c‐MYC, NANOG, SOX2 and OCT4 in chronic myeloid leukaemia (CML) tumour cells. Disrupting the HIF‐mediated hypoxic environment and correcting hypoxic adaptive cell metabolism can reduce the malignancy of tumour cells and enhance drug responsiveness.^94^

The cellular components in the TME also play an important role in protecting CSCs. CAFs maintain the stemness of CSCs by secreting factors such as HGF and promoting EMT.^95^ In addition, the high expression of the ECM receptor integrin on the membrane of CSCs can promote the formation of tight junctions with the ECM, which contributes to physical protection by the ECM and the maintenance of stemness. TACS III shelters CSCs by reducing drug exposure and forming a protective track.

However, according to the important role of tumour stem cells in tumour progression and drug resistance, targeting CSCs in TME could become a promising treatment of cancers.

### 3.2 The TME promotes EMT to initiate metastasis

Metastasis is a sign of tumour deterioration and progression, and a leading cause of cancer death. Tumour metastasis is multistep process regulated by many factors, but EMT is considered the initial step.

EMT occurs when epithelial cells lose their unique polarity, adhesion ability, and surface expression of E‐cadherin and β‐catenin, then assume the morphology of mesenchymal cells and express mesenchymal cell markers such as α‐SMA, vimentin, N‐cadherin and SNAIL.^96^ EMT is involved in normal physiological processes such as embryonic development and wound repair, but also plays an important role in tumour metastasis and CSCs generation. Tumour cells that undergo EMT dysregulate the expression of the myofilaments, microtubules and intermediate filaments that comprise the cytoskeleton, thereby promoting their degradation. These changes allow the cells to lose tight connection with the ECM, obtain higher deformability and motility capacity, and easily break through the tumour basement membrane to pass through gaps between the vascular ECs, and successfully implant in target metastasis organs.^97^ Tumour cells also experience an increase in stemness during EMT.

The TME plays an important role in promoting EMT. TGFβ, EGF, VEGF, HGF and MMP family members in the TME comprise a complex network that promotes EMT. Among those factors, TGFβ is the key EMT‐promoting molecule; it regulates EMT through SMAD‐dependent or independent pathways. In the SMAD‐dependent pathway, TGFβ binds to receptors in the tumour cell membrane; they phosphorylate SMAD family members and promote SMAD2/3 dimer formation. The dimers are transported into the nucleus with the help of activated SMAD4, where they activate a series of EMT transcription factors such as SNAIL, TWIST, vimentin, fibronectin and ZEB.^98^ In the SMAD‐independent pathway, TGFβ activates the MAPK, PI3K/AKT, Wnt/β‐catenin and JAK/STAT signalling pathways in tumour cells, thereby promoting EMT‐related gene expression.^99^

Cells in the TME, especially CAFs, are the main sources of EMT‐promoting molecules. CAFs participate in EMT and tumour metastasis by: (a) secreting paracrine EMT‐related factors,^100^ (b) making direct contact with tumours to promote EMT and (c) mediating matrix remodelling and secreting abnormal ECM collagen and fibronectin. In addition to CAFs, other cells in the TME contribute to EMT. M2 macrophages promote EMT by secreting the immune regulatory factors IL‐6 and IL‐8. Tregs and neutrophils also affect the tumour cell phenotype by up‐regulating the expression of members of the TGFβ pathway.^101^ Various macromolecular proteins in the tumour ECM, such as collagen fibres, are also important for EMT. Breast cancer cells cultured with type I collagen fibres can acquire mesenchymal cell characteristics and undergo EMT. Tumour cells cultured with tumour ECM fibronectin also readily undergo EMT.^102^ High expression of HIF1α in the hypoxic TME inhibits caveolin‐1 expression in tumour cells; a negative feedback mechanism up‐regulates the expression of the caveolin‐1‐related protein epidermal growth factor receptor, thereby activating the STAT3 signalling pathway and reducing the expression of epithelium‐specific markers.^103^ At the same time, mesenchymal transition of tumour‐associated vascular ECs reduces tight junctions, thereby weakening the barrier effects of blood vessels.

However, the current understanding of cellular phenotypical modification during cancer metastasis is acquiring a partial EMT, namely a hybrid E/M phenotype. This indicates that tumours cells can represent mesenchymal phenotype while simultaneously exhibiting epithelial potentials. This hybrid phenotype can be seen in collective invasion of tumour cells, and it accounts for the greatest capacity to pursue tumour metastasis.^104^ These all indicate that targeting EMT activity and weakening the EMT‐promoting link can play a key role in inhibiting tumour metastasis.

## 4 TARGETING THE TME TO BREAK THROUGH THE FORTRESS OF THE TUMOUR

Isolation of tumour cells by the TME results in low treatment efficiencies, low clinical response rates and drug resistance.^13^, ^105^ It has been suggested that highly specific therapeutic strategies that break the barrier of the TME and disrupt its strong protective effects may be safer and more effective.^10^

### 4.1 Targeting drugs attack the TME separately

Understanding the major events occurring in the TME that support primary tumour growth and how these events impact the modulation of the environment is of utmost relevance to assist the definition of efficient therapy strategies. A good deal of current strategies was used to target TME components.

For targeting tumour‐associated ECM, the inhibition of the TGF‐β signalling pathway mediated by Losartan and its analogs results in a reduced secretion of collagen I and consequently reduced ECM stiffness. However, drugs targeting MMPs, include JNJ0966, highly selective towards MMP‐9, and the antibody Fab 3369 that targets MMP‐14, have been developed. Among them, incyclinide went through several clinical trials for advanced carcinomas (Clinical trials NCT00004147, NCT00003721, NCT00001683 and NCT00020683).^106^ Drugs targeted against CAFs have come into clinic therapy. Nindetanib or BIBF1120 having tyrosine kinase inhibitor activity towards receptors of VEGF, FGF and PDGF greatly reduce the protective effect of CAFs on tumour. Several antiangiogenic drugs have been tested in clinical trials. Monoclonal antibodies of VEGF/VEGFR, such as bevacizumab and pazopanib, increased overall survival or progression‐free survival of patients when used alone or as adjuvants in a cocktail chemotherapeutic treatment.^107^ In addition, targeting immune system in TME also play an important role in tumour treatment. The inhibition of colony‐stimulating factor‐1 (CSF‐1) signalling by anti‐CSF‐1R neutralizing antibodies has been shown to impair TAMs‐recruiting and MDSCs mediated tumour proliferation. 28117416 Activation of the anti‐tumour functions of the immune cells by GM‐CSF, which stimulates the antibody‐dependent cellular cytotoxicity of anti‐cancer antibodies, and targeting of immune checkpoint inhibitors (CTLA‐4, PD‐1, PD‐L1) has been widely exploited to prevent tumour progression.^108^

### 4.2 Cell therapy to modify the TME is a new strategy for tumour treatment

**FIGURE 3:**
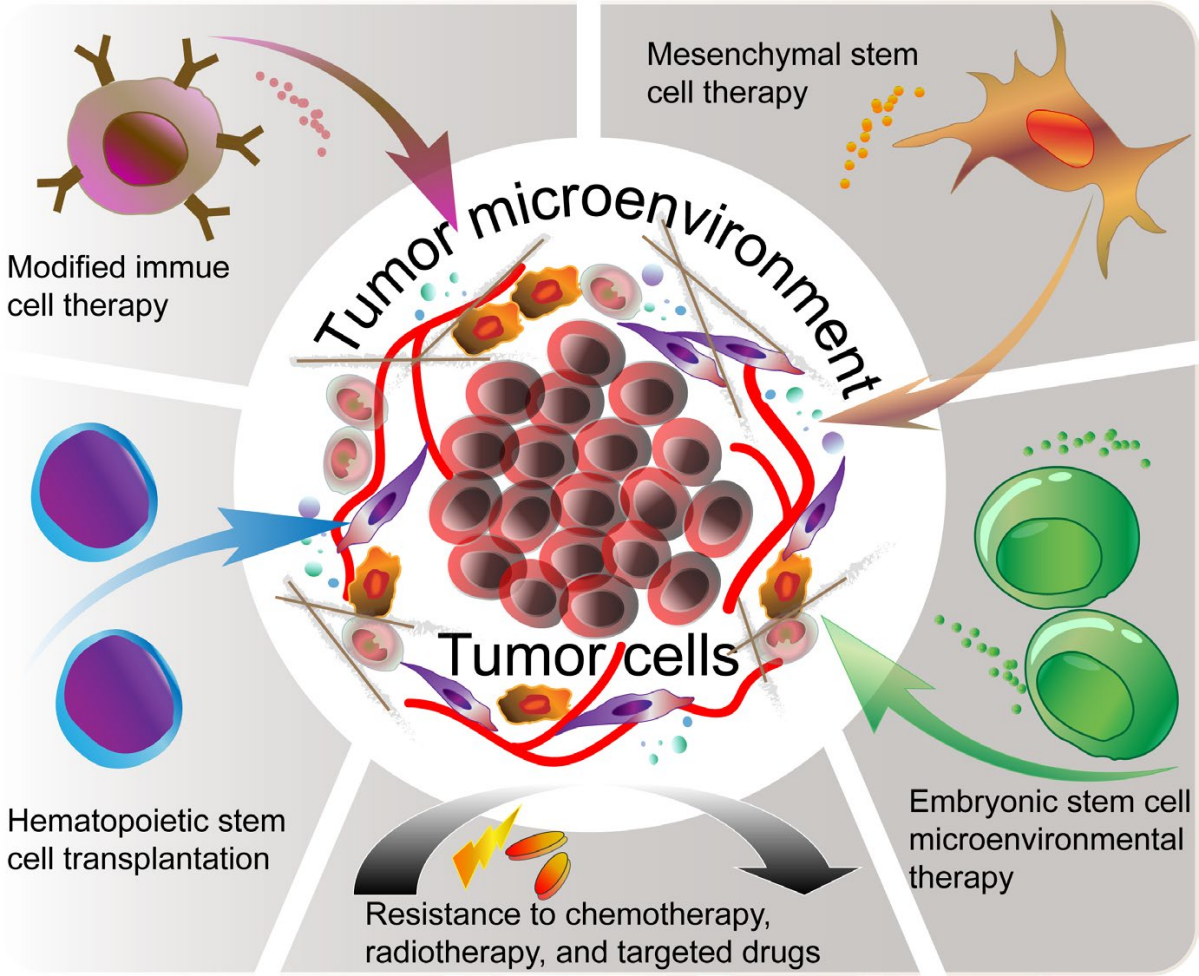
Cell therapy to modify the TME. Traditional radiotherapy, chemotherapy and targeted drugs all rely of tumour cell killing as their main mechanism of action, with the attendant serious adverse reactions and rapid resistance. Current cell therapies for targeting tumours and the TME—modified immune cell therapies, haematopoietic stem cell transplantation, mesenchymal stem cell transfer, and embryonic stem cell‐based microenvironment therapies—provide novel ideas for exploring breakthrough of tumour therapy strategies

Even though targeting molecules inhibit tumour to a great extent, there is still some insurmountable difficulties. For example, agents that degrade and/or deconstruct ECM must be used carefully, since they may induce metastasis instead of avoiding tumour progression. We boldly assume that cell therapies may solve some of the problems of targeting drugs attributing to cell's natural advantages. (a) The cell itself has good plasticity, which provides the possibility of acting on multiple TME components at the same time to fully degrade TME, which can effectively prevent the compensatory regeneration and protection of other components when simply targeting a certain part; (b) the cell has homing properties. The "localization system" of the treatment cells has the tendency of tumour tissue and homing under the action of cytokines and chemokines, which greatly avoids the systemic side effects brought by non‐selective drugs; (c) cell has high permeability. Due to tumour stiffness and abnormal angiogenesis, the drug's permeability to TME is poor, but cells can effectively enter TME through deformation movements, secretion of matrix‐regulating enzymes, etc; (d) many cells have the ability to regulate normal tissue regeneration and repair, which can correctly repair tissue damage caused by chemotherapy drugs and avoid the adverse impact of bystander effects on normal cells (Figure 3).

#### 4.2.1 Immune cell therapy for precisely targeting tumour cells

The TME regulates the differentiation and activation of immune cells to reduce their ability to recognize and eliminate tumour cells. Immune cell therapies that more accurately target tumour cells while restoring normal immune cell populations within the TME, enhancing normal immune cell function and disrupting the protective effects of the abnormal immune cell networks have become a new anti‐tumour option.^109^ As early as 1998, studies demonstrated that transplanting immune‐activated splenocytes into mice with breast cancer caused tumour shrinkage and conferred long‐term tumour‐free survival, suggesting that adoptive immune cells can be effective anti‐tumour therapeutics.^110^ In recent years, immune cells such as NK cells, cytokine‐induced killer cells, DCs, tumour‐infiltrating lymphocytes and chimeric antigen receptor (CAR)‐modified immune cells have shown marked anti‐tumour potential.^111^ Among them, CAR‐modified cells have the best effects and have gradually made the transition from scientific research products to clinical treatment option.

A CAR is a biological element that is transgenically expressed on the surface of an immune cell membrane where it recognizes specific antigens. The extracellular structure of a CAR is a variable single‐chain fragment that recognizes a specific antigen. The intra‐membrane structure comprises a signal transduction domain; the earliest CARs used a single CD3ζ domain, but successive generations have included different numbers and types of co‐stimulatory domains to improve the cell proliferation, cytokine secretion and killing capacities of CAR‐equipped immune cells.^112^ CAR‐modified immune cells are purified and expanded from autologous or allogeneic immune cells or stem cell‐derived cells, and then engineered with a CAR and transferred into patients. They have a lower risk of mediating off‐target graft‐versus‐host responses while killing tumours. Upon recognition of tumour cells that express their target antigens, CAR‐modified cells activate pathways to initiate their proliferation, release of inflammatory factors and phagocytosis to kill the targeted cells accurately and specifically. At present, the most commonly CAR‐modified immune cells are CAR‐T and CAR‐NK cells, which have achieved good responses in the clinical treatment of haematological tumours.^113^ Many groups have explored the feasibility of applying these tools to solid tumours. As of 2018, 57% of CAR‐T‐cell clinical trials focused on the treatment of haematological malignancies, 11% on gastrointestinal tumours, 8% on neurological tumours and 5% on breast cancers.^114^

CAR‐T cells are currently the most mature anti‐tumour immune cells. Among these, CAR‐T cells targeting CD19 have been the most successful in the clinic. A clinical trial at Memorial Sloan‐Kettering Cancer Center in 2016 (NCT01044069) showed that 91% of 32 B cell‐derived acute lymphoblastic leukaemia (B‐ALL) patients treated with CD19‐targeted CAR‐T cells achieved complete remission. Another clinical trial (NCT01626495) demonstrated that 30 patients with B‐ALL had a complete response rate of 90% after CD19 CAR‐T‐cell therapy. In a National Institutes of Health clinical trial in 21 children and adults with ALL (NCT01593696), a complete response rate of up to 70% has been reported. CD19‐specific CAR‐T cells also show limited therapeutic effects on other haematologic malignancies such as chronic lymphocytic leukaemia, lymphoma and multiple myeloma (NCT02135406); however, the response rates of these diseases are lower than that of B‐ALL.^115^, ^116^ CAR‐T cells targeting other molecules, such as CD20, CD22, CD30 and ROR1, have been used successfully to target different tumours (NCT02315612 and NCT00621452).^117^ In addition to blood tumours, many studies have tried to target solid tumours using CAR‐T cells, such as GD2 CAR‐T for neuroblastoma and GPC3‐targeted CAR‐T cells for hepatocellular carcinoma, which have prolonged patient survival.^118^, ^119^ However, CAR‐T is highly effective for malignancies of haematologic system; for solid tumours, the application of this strategy is elusive mainly because of their suppressive TME, weak TIL trafficking,^120^, ^121^ and has a high probability of side effects. In clinical trials of CD19‐specific CAR‐T‐cell treatment, all subjects without exception showed cytokine release syndrome. In addition, complications such as neurotoxicity, B‐cell depletion and off‐target effects also seriously threaten patients' lives.^122^

In addition to CAR‐modified T cells, CAR‐NK cells have achieved a high response rate in the treatment of myelodysplastic syndrome and acute myeloid leukaemia (AML). CAR‐NK cells for the treatment of B‐cell lymphomas (NCT01974479 and NCT03056339) and metastatic solid tumours (NCT03415100) are in clinical trials; however, they face problems such as low CAR transfection efficiency and short in vivo survival time.^123^ To resolve the issues of limited sources of NK cells and short survival time, NK cells have been generated from induced pluripotent stem cells (iPSCs) and cord blood, and their therapeutic effects on recurrent or refractory blood and solid tumours have been assessed (NCT01729091, NCT03019640, NCT02280525 and NCT03539406). However, the low induction and differentiation efficiencies of iPSCs and the relatively high risk of tumour formation still limit their application.

Exosomes secreted by CAR cells have been confirmed to carry the CAR structure. They maintain the original targeting characteristics of the parent cells in the context of lower off‐target cytotoxicity, with high efficacy and safety in preclinical experiments.^124^ CAR exosomes may be used to optimize or even replace existing cell‐based CAR immunotherapies.

#### 4.2.2 Haematopoietic stem cell transplantation to reconstruct haematopoietic function

The origins of many tumours are associated with the dysplasia, depletion, and dysregulated proliferation and differentiation of normal stem cells. The existence of a normal stem cell microenvironment has become a prognostic indicator for tumour treatment.^125^ Eliminating malignant CSCs and supplementing with normal stem cells has become a reliable treatment for many tumours, especially haematologic tumours.^126^

Haematopoietic stem cell transplantation (HSCT) is to completely remove the abnormal bone marrow haematopoietic stem cell (HSC) microenvironment by means of radiotherapy and chemotherapy, and then rebuild the haematopoietic microenvironment and restore the normal haematopoietic function by transplanting normal stem cells. As the only curative treatment for malignant and non‐malignant diseases of the haematopoietic system, HSCT has developed rapidly in the past 25 years.^127^, ^128^, ^129^, ^130^ The first step of HSCT is the pretreatment of patients with radio‐chemotherapy to destroy the dysfunctional haematopoietic and immune systems and to ensure low immune responsiveness by the patients, thus laying the foundation for long‐term survival of the transplanted cells. Then, HSCs—mobilized from the donor by granulocyte‐colony‐stimulating factor—are purified, expanded in vitro, and transplanted into the recipient, where they typically home to the bone marrow and gradually reconstitute the recipient's haematopoietic system. HSCT brings graft‐versus‐leukaemia (GVL) effect in recipients, which helps eradicate tumours and is considered to be multifactorial.^131^ The mechanism of GVL, though poorly understood, might be similar to graft‐versus‐host disease (GVHD) phenomenon that is mediated by donor's immune compounds. The principal cytotoxicity in GVL is mediated by donor T cells and NK cells with ancillary roles played by dendritic cells, B cells and minor histocompatibility antigens.^132^ Although HSCT can fundamentally eradicate tumours, several side effects, including acute infection, graft rejection, chronic GVHD and secondary tumour development after transplantation, still impair the long‐term survival of patients.^133^, ^134^ Approximately 1.2%‐1.6% of patients have secondary tumours 5 years after HSCT, and the cumulative incidence of secondary tumours increases to 2.2%‐6.1% after 10 years, and 3.8%‐14.9% after 15 years.^135^ To reduce the side effects and improve the survival rates of patients, HSCT programmes have continuously innovated. Peripheral blood‐ and umbilical cord blood‐derived HSCs are gradually replacing bone marrow HSCs for transplantation, which solves the problem of insufficient cells.^136^ HSCT performed under non‐myelosuppressive conditions by reducing the doses of chemotherapy and radiation, or even without pretreatment, reduces the incidence of the GVHD response and improves long‐term transplantation efficacy.^137^ It is clear that maximizing the GVL effect while minimizing GVHD is the holy grail of transplant immunology. Given that GVHD and GVL have similar mechanisms, prophylaxis for GVHD might affect GVL intensity, which should be taken into account when formulating a transplantation strategy.

HSCT is becoming increasingly prominent in the treatment of some solid tumours, especially refractory tumours.^138^ As early as 1997, researchers found that breast tumours in mice shrank after allogeneic HSCT.^139^ In recent years, HSCT has been studied for the treatment of relapsed and refractory glioma, and as a consolidation therapy for the remission stage, resulting in a 1‐year tumour‐free survival rate of up to 90%–93% and a 3‐year survival rate of 63.7%.^140^, ^141^ After receiving allogeneic HSCT, especially non‐myelosuppressive transplantation, patients with metastatic renal cell carcinoma showed a relatively high response rate, likely due to the restoration of T cells and other immune cells capable of disrupting the immunosuppressive TME.^142^, ^143^

#### 4.2.3 MSC therapy

MSCs are non‐haematopoietic stem cells primarily found in the bone marrow. They can differentiate into adipose tissue, bone and cartilage under the appropriate conditions.^144^ In addition to the bone marrow, MSCs also exist in cord blood, peripheral blood and adipose tissue.^145^ Due to their relative abundance, shared features with tumours and homing characteristics, MSCs can effectively enter the TME, thereby overcoming low drug delivery efficiency and limitations on TME penetration.^146^ Transfer of pre‐edited and modified MSCs has become a new strategy for tumour cell therapy.^147^

The reported effects of natural MSCs on tumours have been contradictory. Some studies have shown that MSCs have anti‐tumour effects, such as inhibiting tumour cell proliferation and causing cell cycle arrest in leukaemia, bladder cancer, renal cell carcinoma, breast cancer and other tumours.^148^, ^149^, ^150^, ^151^ However, other studies have found that MSCs promote tumour progression. MSC transplantation can boost tumour invasiveness. This is due to that the cells highly express CXCR4, upon injection, these CXCR4 + cells are attracted into the areas enriched in CSCs or cancer stem niches. In these places, MSCs can alter their phonotypical characteristics into attaining a CSCs like feature, thus enriching the pool of cancer stem niches for the subsequent progression and metastasis of tumours like glioblastoma.^152^ Relevant researches show that osteosarcoma cells have enhanced proliferation and migration ability after co‐culture with MSCs in vitro^153^; MSCs enhance the thermal resistance of ovarian cancer cells through CXCL12‐dependent pathways and promote tumour development and angiogenesis in mice with colon cancer^154^, ^155^; With support from MSCs, breast cancer cells acquire high metastasis ability through the CCL5/CCR5 feedback axis.^156^, ^157^ MSCs may secrete pro‐tumour chemokines and differentiate into CAFs in the TME. Studies demonstrated that effect of MSCs is opposite within different injection site in the same animal model. When injected locally with breast cells, MSCs promoted the migration and invasion abilities of tumour cells, and drove the angiogenesis progress. To the contrary, distant injection of MSCs inhibited tumour progression and promoted altered immune cell populations in the TME. Treg and MDSC were decreased significantly, and CD8 T cells and DCs were increased. Immune‐activating cytokines TNF, IFNγ, TLR3 and IL‐12 were up‐regulated. These results imply we should pay attention to the administration methods when apply MSCs to tumour patients.^158^, ^159^ We hypothesize that MSCs could concurrently behave pro‐tumour effects by differentiating into CAFs, pro‐angiogenesis, and inhibit T cells to enhance TME compounds, while tend to suppress tumours by activating innate and adaptive immune, the final effect depends on the order and strength of the two sides and tumour context.

MSC exosomes, engineered MSC nanoparticles (nanoghosts), and other microvesicles have been used as substitutes for MSCs due to their similar compositions and better safety profiles. Exosomes derived from MSCs can inhibit the proliferation of various tumour cells.^160^, ^161^, ^162^ However, there also have been reports that MSC exosomes promote tumours.^163^ The best method to make use of the advantages of MSCs while avoiding their tumour‐promoting activity has become the focus of MSC therapy research.

Researchers are increasingly modifying MSCs to optimize their anti‐tumour function in 2 ways. First, they are enhancing the synthesis and release of endogenous and exogenous anti‐tumour factors from MSCs. Second, they are strengthening their homing to the TME and prolonging their activity there to achieve more efficient TME penetration and anti‐tumour effects.

In order to ensure the tumour‐killing activity of MSCs and prevent them from promoting tumours, many researchers have enhanced MSC synthesis and release of IFN‐γ, which inhibits tumour cell proliferation, and tumour necrosis factor‐related apoptosis‐inducing ligand, which promotes apoptosis.^164^, ^165^ These approaches have enhanced MSC anti‐tumour effectiveness in preclinical and clinical experiments. MSCs transfected with suicide genes and carrying biologically active anti‐tumour substances reach the TME via recruitment by chemotactic signals such VEGF and TGFβ1 secreted by tumour cells and CAFs. Once in the TME, they initiate the suicide programme to release the drugs with greater specificity and in greater concentration in the TME than achievable by conventional methods.^166^, ^167^, ^168^ These findings suggest the feasibility of using modified MSCs as efficient drug carriers. MSCs transfected with only a CCL5 promoter‐driven or ganciclovir‐induced suicide gene can also cause a degree of tumour cytotoxicity in animal models and clinical trials of hepatocellular carcinoma, pancreatic cancer, and breast cancer.^169^, ^170^

In order to enhance the in vivo activity of MSCs, researchers have encapsulated MSCs with synthetic biodegradable ECM and implanted them in the brains of mice with glioblastoma. This approach resulted in dramatic shrinkage of the tumour and ensured the long‐term biological activity of the MSCs.^171^ Other studies have enhanced the homing of MSCs through gene editing. HIF1α induced by the hypoxic TME up‐regulates downstream SDF‐1α to promote tumour proliferation and metastasis. Overexpression of the SDF‐1α receptor CXCR4 in MSCs enhances the penetration of MSCs into the hypoxic glioma microenvironment.^172^

However, MSCs remain a double‐edged sword. The stronger the effects of MSCs as drug carriers that penetrate the TME to kill tumours, the more serious the side effects caused by the drugs' non‐selective destruction of normal cells. The current drawbacks associated with MSC therapies have prompted researchers to seek safer, more accurate anti‐tumour treatment methods.

#### 4.2.4 Embryonic stem cell microenvironmental therapy

The early embryonic microenvironment has the powerful ability to repair erroneous genetic material and inhibit oncogene expression; thus, embryonic cells have innate tumour immunity characteristics. An increasing amount of research is being devoted to applying this tumour‐hostile microenvironment to tumour therapy.^173^, ^174^

The embryonic microenvironment can reverse tumour fate. Lee et al^175^ implanted human skin melanoma cells into zebrafish blastocysts and, surprisingly, found that the implanted cells did not form tumours in the embryo. Kasemeier‐Kulesa et al^176^ found that malignant melanoma cells implanted into chicken embryos did not produce tumours, and even reverted to expressing the normal melanocyte‐specific marker MART‐1. Durr et al did not observe tumour formation after they implanted human KG‐1 myeloid leukaemia cells and primary human AML cells into 3.5‐day (mulberry phase) mouse blastocysts. The tumour cells implanted into the embryonic microenvironments reverted to the expression of erythroid‐specific haemoglobin and glycophorin A, and resumed proper erythroid homing to the aorta‐gonad‐mesonephros region (embryonic haematopoietic tissue), the yolk sac, and the peripheral blood.^177^ The ability of the embryonic microenvironment to revert tumour cells gradually weakens as the embryo develops and differentiates.^178^ (Table 1).

**TABLE 1:** Summary of studies on the ability of the embryonic microenvironment to reverse the malignant tumour phenotype

Mimic embryonic microenvironment	Target diseases	In vivo administration route	Mechanisms	Reference
hESC‐conditioned medium/hESC‐ exosomes	Colorectal adenocarcinoma, breast cancer	Subcutaneous injection	Rebalance ectopic expression of OCT4, SOX2, KLF and c‐MYC	173
mESC‐co‐culture	Uveal melanoma	Subcutaneous injection	Down‐regulation of PI3K/AKT pathway	180
mESC‐intravenous injection	Chronic myeloid leukaemia	Intravenous injection	NA	179
mESC‐conditioned medium	Breast cancer	Mammary fat pads injection	Down‐regulation of STAT3 pathway	183
hESC‐conditioned medium	Colorectal carcinoma	Subcutaneous injection	Suppress Notch1 pathway	184
3D decellularized matrix after hESCs culture	Cutaneous melanoma, breast cancer	Mammary fat pads injection	Down‐regulation of nodal pathway	178

Abbreviations: hESC, human embryonic stem cell; mESC, mouse embryonic stem cell; TCTP, translationally controlled tumour protein.

In order to apply the powerful tumour‐reverting effects of the in situ embryonic microenvironment to the practical treatment of tumours, researchers have cultured embryonic stem cells (ESCs) in vitro to establish an ESC microenvironment (ESCM) that mimics that found in embryos with remarkable results. Zhou et al injected TK suicide gene‐transfected ESCs into mice with CML via the tail vein and found that the cells restored normal blood cell counts in the mice and prolonged their survival. By timely activation of the suicide gene, the differentiated ESCs could be eliminated to avoid teratoma formation and guarantee the safety of the recipients.^179^ Liu and Wang et al co‐cultured embryonic stem cells with uveal melanoma or cutaneous melanoma cells. They found that the ESCM can inhibit malignant phenotype development in tumour cells. Upon co‐culture of ESCs, healthy cells and tumour cells, the ESCM surprisingly down‐regulated the PI3K pathway in the tumours, but up‐regulated the PI3K pathway in normal cells. Thus, the ESCM played a bidirectional regulation role by reversing tumour‐related signalling and enhancing signalling in healthy cells. This finding proved that killing tumours does not inevitably result in off‐target killing of normal cells.^180^, ^181^

Other research has confirmed the tumour‐reverting effects of the ESCM. For example, decellularized matrix components generated by 3‐dimensional ESC culture can reverse metastatic breast and prostate cancers.^176^ Furthermore, human ESC‐conditioned medium and exosomes can suppress the malignant phenotypes of colon cancer and breast cancer cells. The researchers speculated that the effects of the ESCM on tumour reversion may result from the exchange of materials between cells, thereby rebalancing the expression of the stemness factors OCT4, SOX2, KLF and c‐MYC in tumour cells.^173^ Zebrafish embryo extracts can inhibit breast cancer by down‐regulating the expression of translationally controlled tumour protein and promoting E‐cadherin/β‐catenin redistribution to reshape the cytoskeleton.^182^ Mouse ESC‐conditioned medium was found to inhibit breast cancer cell proliferation, promote apoptosis and inhibit malignant behaviour.^183^

The mechanisms by which the ESCM reverts tumours are still not completely clear. Current studies suggest that tumour reversion may be related to the PI3K/AKT, STAT3, Notch1, and other pathways, and that exosomes derived from ESCs may play an important role in their signal transduction.^178^, ^183^, ^184^ In addition, transmembrane communication and cell contact‐mediated signal transduction may play key roles in ESCM regulation of tumour cell fate. The totipotency of ESCs enables them to regulate the TME in many ways, not only by affecting tumour cells, but also by reshaping other TME components, enhancing normal cell functions, and supporting the regeneration and repair of the body. ESC treatment raises new hope for radically eliminating the side effects caused by killing normal cells during tumour treatment.

## 5 SUMMARY AND PROSPECTS

The TME can mediate tumour cell immune escape, promote CSCs formation and enhance tumour metastasis ability, thereby promoting tumorigenesis and development. The distinctive features of the TME, including the abnormal haemodynamics due to neovascularization, the ECM that is difficult to penetrate, and the hypoxic state that confers resistance to oxidative damage, play important roles in the drug resistance of tumour cells. Cell therapies utilizing engineered immune cells have shown good anti‐tumour effects, but poor curative effects for solid tumours. Furthermore, the main aim of this type of cell therapy is still to kill tumour cells. Thus, the body can develop resistance to the treatment, and it is impossible to avoid the complications caused by cell killing that are also associated with traditional therapy. Malignant tumour cells are derived from normal cells and develop in the TME. Suitable microenvironments such as the early embryonic environment can revert tumour cells into normal cells. HSCT, MSC transplantation, and ESC and other stem cell therapies can reach the interior of the TME due to their strong targeting and penetrating power. By reshaping the cellular components, ECM structure and soluble factor composition to comprehensively rebuild the TME, these stem cell therapies can reverse the malignant tumour phenotype while avoid cell killing and reducing treatment‐related harm to patients. Stem therapies could, therefore, represent the most promising new direction for tumour treatment. New breakthroughs in tumour treatment await in the near future based on combining approaches to complement the strengths of individual cell therapies, optimizing the favourable characteristics of cells with engineered modifications to compensate for their shortcomings, or replacing source cells with cell‐specific microvesicles.

## CONFLICT OF INTEREST

The authors declare no conflict of interest.

## AUTHOR CONTRIBUTIONS

CYQ, SBW and JHL wrote the first draft of the manuscript. JL, TL, CYL, YRS and YRL wrote sections of the manuscript. CYQ, XS, QW participated in the discussion of the manuscript. CL, LY and ZCW provided critical revisions. All authors approved the final version of the manuscript for submission.

## Data Availability

Data available on request.
